# Efficacy of Game-Based EMG-Biofeedback Therapy in Post-Stroke Dysphagia: A Randomized Controlled Trial

**DOI:** 10.1007/s00455-025-10819-1

**Published:** 2025-03-05

**Authors:** Bülent Alyanak, Murat İnanır, Selime Ilgın Sade, Serkan Kablanoğlu

**Affiliations:** 1 Department of Physical Medicine and Rehabilitation, Kocaeli University Faculty of Medicine, Kabaoğlu, Baki Komsuoğlu Bulvarı No:515, Umuttepe, 41001 İzmit, Kocaeli Turkey; 2 Institute of Health Sciences, Kocaeli University, Kabaoğlu, Baki Komsuoğlu Bulvarı No:515, Umuttepe, 41001 İzmit, Kocaeli Turkey

**Keywords:** Biofeedback, Dysphagia, EMG, Game, Rehabilitation, Stroke

## Abstract

The aim of this study was to investigate the effects of game-based electromyography (EMG)-biofeedback therapy on swallowing functions and quality of life in patients with post-stroke dysphagia. This prospective, assessor-blind, randomized controlled trial included 33 patients with post-stroke dysphagia. The study group performed the Mendelsohn maneuver and effortful swallow using game-based EMG-biofeedback, while the control group performed the same tasks with only verbal feedback for 30 min across a total of 15 sessions. In addition, both groups received oral motor exercises and thermal-tactile stimulation for equal durations. Patients were evaluated immediately before and after the treatment using clinical swallowing assessments, including the Functional Oral Intake Scale (FOIS), Penetration-Aspiration Scale (PAS), Dysphagia Outcome and Severity Scale (DOSS), and Dysphagia Handicap Index (DHI). Statistically significant improvements were observed in FOIS scores (p = 0.038), PAS-Liquid scores (p = 0.026), and DOSS scores (p = 0.003) in the study group, while no significant changes were noted in the control group. PAS-Semisolid scores improved in both groups (study group, p = 0.002; control group, p = 0.023); however, post-treatment scores were significantly higher in the study group compared to the control group (p = 0.031). Although significant improvements in DHI total, physical, emotional, and functional scores were observed in both groups at the end of treatment (all p < 0.05), post-treatment DHI scores were significantly higher in the study group compared to the control group (all p < 0.05). The addition of game-based EMG-biofeedback to conventional therapy improved clinical and radiological outcomes, as evidenced by improvements in FOIS, PAS-Liquid, and DOSS scores, and led to statistically significant improvements in PAS-Semisolid and DHI scores. In conclusion, the inclusion of game-based EMG-biofeedback therapy in swallowing rehabilitation programs may have a positive impact on treatment outcomes.

## Introduction

Stroke is the second leading cause of death and the third leading cause of disability in the world according to the Global Burden of Disease study conducted in 2019 [1]. Stroke has many serious life-threatening complications, of which dysphagia is one of the most severe. Dysphagia is observed in 20–70% of stroke patients in the acute period, 10–40% in the subacute period, and 5–25% in the chronic period [2]. These rates depend on the type of stroke, its severity, individual characteristics, and the type of diagnostic assessment used to detect dysphagia and the time elapsed until the assessment [3]. Patients with post-stroke dysphagia may face complications such as malnutrition, dehydration, weight loss, aspiration, and even death. These complications increase duration of hospitalization and associated costs and reduce quality of life [4]. Additionally, they adversely affect patients' participation in rehabilitation and rehabilitation outcomes.

Various treatment methods are employed for dysphagia rehabilitation including postural changes, altering food viscosity, swallowing maneuvers, therapeutic exercises, sensory stimulation, repetitive transcranial magnetic stimulation (rTMS), neuromuscular electrical stimulation (NMES), pharyngeal electrical stimulation (PES), and transcranial direct current stimulation (tDCS) [5, 6]. However, despite their widespread use, these treatment methods have certain limitations. For instance, postural changes and food viscosity modifications may offer temporary relief but do not address the underlying pathophysiology of dysphagia. Similarly, sensory stimulation and neuromodulation techniques like rTMS or tDCS often require specialized equipment and expertise, which may limit their accessibility. Additionally, the efficacy of therapeutic exercises heavily depends on patient adherence and proper technique. In light of these limitations, the growing interest in EMG-biofeedback therapy has emerged as a promising approach. The growing interest in EMG-biofeedback therapy may be attributed to its potential to actively engage patients in the rehabilitation process and improve treatment outcomes.

Surface electromyography (sEMG) is the most commonly used biofeedback method as a tool to record swallowing movements and provide feedback. sEMG provides real-time biofeedback on quantitative muscle electrical activity, which promotes patients' awareness and control of muscle activation and relaxation and can improve motor accuracy and coordination during exercises [7]. It also increases motivation and treatment efficacy by allowing individuals to self-monitor their performance during monotonous swallowing exercises [8]. Game-based application of EMG-biofeedback can provide the patient with stronger feedback both audibly and visually, making the treatment more enjoyable, thereby increasing the patient's motivation towards the goal and enhance treatment participation and effectiveness [9]. Numerous studies provide evidence that an extrinsic focus of attention accelerates the learning process so that higher levels of efficiency and skill are reached more quickly [10]. Moreover, a consistently high level of exercise intensity during this process can help trigger further neuroplastic changes [11, 12]. A functional magnetic resonance imaging (MRI) study showed that swallowing activity with biofeedback increases cortical activation [13].

Although there are numerous studies applying EMG-biofeedback in dysphagia treatment, these studies vary methodologically, and the number of randomized controlled trials is quite limited. Two systematic reviews and meta-analyses have concluded that studies examining the effectiveness of biofeedback therapy in dysphagia due to different etiological factors are of low to moderate methodological quality [8, 14]. Furthermore, many of these studies involve small patient populations or fail to use gold-standard methods for outcome assessment.

Based on the hypothesis that compared to conventional practices, rehabilitation interventions accompanied by game-based EMG-biofeedback may be more effective in improving swallowing functions in patients with post-stroke dysphagia, the aim of the present study was to prospectively and systematically demonstrate the effects of exercise interventions using game-based EMG-biofeedback on swallowing functions and quality of life.

## Methods

### Study Design

This study was designed as a prospective assessor-blind randomized controlled trial and was conducted between April 2023 and October 2023. The inclusion and exclusion criteria are presented in Table 1.

**Table 1:** Inclusion and exclusion criteria of the participants

Inclusion criteria:	
- History of hemorrhagic or ischemic stroke lasting more than 3 months	
- Age of 18 years or older	
- Onset of swallowing complaints after a stroke	
- Mini-Mental State Examination (MMSE) score of at least 24	
- Absence of serious concomitant systemic illnesses (uncontrolled hypertension, decompensated heart failure, malignancy, infection, cardiac pacemaker, epilepsy, tracheostomy, etc.)	
- Functional Oral Intake Scale (FOIS) score of ≤ 6	
- Detection of pathology in the oropharyngeal phase of swallowing during videofluoroscopic evaluation	
- No treatment related to dysphagia in the last 3 months	
Exclusion Criteria:	
- History of neoplastic disease and/or radiation therapy or surgery to the head and neck region	
- Presence of additional musculoskeletal or neurological diseases outside of stroke that could cause swallowing disorders	
- Inability to communicate or follow commands	
- Inability to maintain head stability	
- Presence of significant pathology in the formation of the bolus in the oral phase of swallowing or during the passage of the bolus to the pharynx	

Patients were randomized into two groups by simple random sampling method through a computer program: EMG-Biofeedback (study) and conventional therapy (control). Patients' demographic information, medical and family history, time elapsed after stroke, radiological findings, stroke type, lesion location, type of nutrition, medications used, and post-stroke treatments were recorded. Additionally, MMSE, Functional Ambulation Scale (FAS), Barthel Index, and Gugging Swallowing Screen (GUSS) scores were recorded. FOIS, PAS, DOSS, and DHI were administered to the patients in both groups before and after therapy. To ensure the conditions of assessor blinding, evaluations were conducted by another researcher who was blinded to the intervention and unaware of the group allocation of the individuals, both before and after therapy. All extracted data were analyzed by a data analyst who was also blinded to the study.

### Intervention

#### Conventional Therapy

##### Oral Motor Exercises and Thermal Tactil Stimulation

Both groups received 15 min of oral motor exercises and 10 sessions of thermal-tactile stimulation, each lasting approximately 5 min, administered daily throughout the treatment period.

##### Effortful Swallow

All participants were taught the effortful swallow. They were instructed to push the hard palate strongly with their tongue, squeeze all the throat muscles, and swallow forcefully. To facilitate learning, the patients were told to "swallow hard, as if swallowing something stuck in your throat." The maneuver was practiced several times to ensure that the patients performed it correctly.

##### Mendelsohn Maneuver

Patients’ laryngeal prominence (thyroid notch) was palpated, and the patients were asked to palpate it as well. Patients were instructed to place their index finger on the laryngeal prominence, swallow, and hold the laryngeal prominence in the highest position for 2–3 s without allowing it to drop. It was emphasized that breathing would stop during this time, and if they could breathe, they were doing it wrong. The patients were given several practice attempts to ensure proper execution of the exercise. During the treatment period, they were encouraged to hold the position for a longer duration, and for those able to do so, this duration was extended to 5 s.

Patients in the control group performed the Mendelsohn maneuver and the effortful swallow for 15 min each for a total of 30 min with only verbal feedback such as "hold longer" and "squeeze harder." The study group practiced these exercises with game-based EMG-biofeedback. The total duration of sessions for both groups was kept equal. Both groups received treatment for a total of 15 sessions over a period of 3 weeks, with sessions held 5 days a week.

Patients with sitting balance were seated on a chair. For patients unable to sit, their beds were adjusted to an upright position, and exercise therapy was commenced. Both groups were given 2 ml of liquid to moisten the oropharynx during exercises when needed, and for patients at risk of aspiration, the oral cavity was moistened with moist gauze. Short rest periods were provided for patients during exercise sessions.

### Game-Based EMG-Biofeedback

**Fig. 1:**
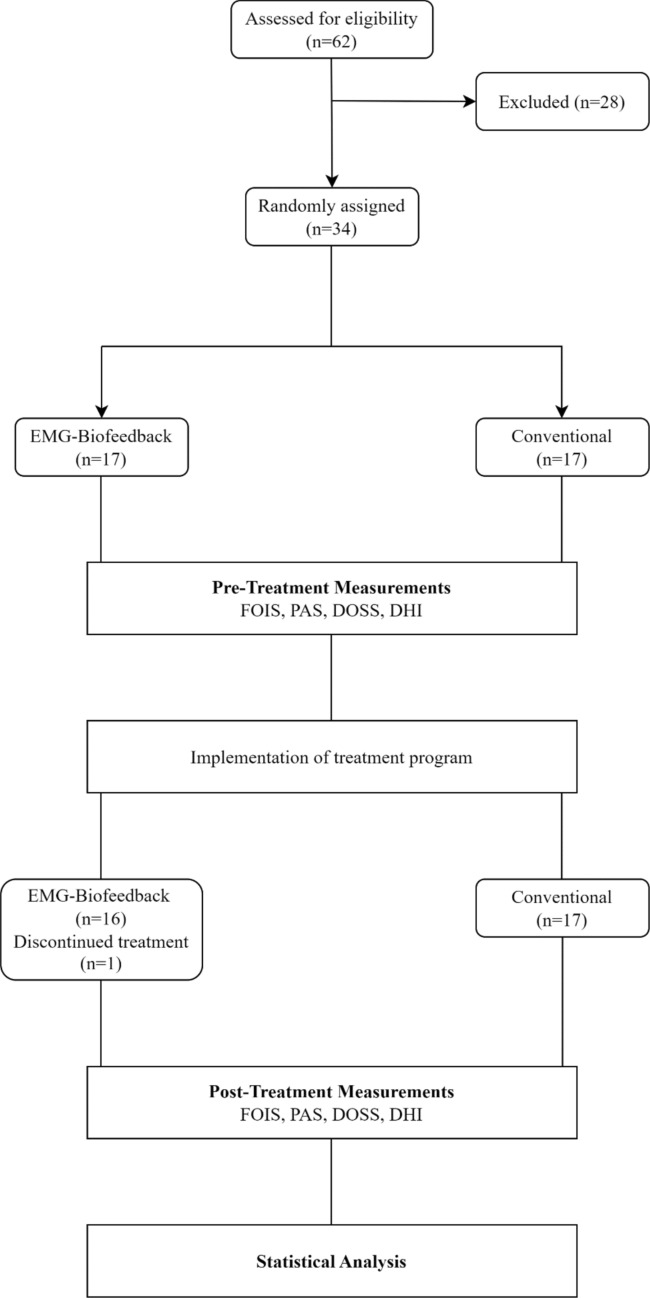
The electrode placed horizontally on the submental region (suprahyoid muscles)

Patients included in the study group performed effortful swallow and Mendelsohn maneuvers with game-based EMG-biofeedback. Game-based EMG-biofeedback was applied using the Vitalstim Plus device (Chattanooga Group, Hixson, TN, USA) and the Vitalstim Software obtained from the manufacturer's official website. Before electrode placement, the skin was prepared by cleaning with chlorhexidine/alcohol wipes. The bipolar surface electrode was placed horizontally over both sides of the patient's submental muscles (mylohyoid, geniohyoid, anterior digastric), equidistant from the midline, and the reference electrode was secured under the clavicle. sEMG was performed using a Vitalstim Plus device (Chattanooga Group, Hixson, TN, USA) with a selectable band-pass filter, a heart rate filter with a bandwidth of 100–370 Hz with the heart rate filter turned on, and a 50 Hz notch filter, with a root mean square (RMS) range of 0.2 to 2000 μV and a sensitivity of 0.1 μV RMS, and the measurements were simultaneously transferred via Bluetooth to the Vitalstim Software. EMG-biofeedback was applied with the rose game and bunny game available in the Vitalstim Software program on the computer screen. The games provided patients with both visual and auditory feedback (Fig. 1).

''Effortful swallow'' was performed with the rose game and ''Mendelsohn maneuver'' was performed with the rabbit game. In the effortful swallow, the patients were asked to press their tongue hard against their palate and swallow with all their strength. Meanwhile, the rose on the screen would fade when the patients exceeded a threshold in muscle activity. Once the patient completely faded the rose, indicating the completion of the swallowing activity, they would relax their muscles, and the rose would bloom again, completing one exercise cycle. The game provided not only visual but also auditory feedback at certain stages. In the rabbit game, the patient was visualized as a rabbit climbing a hill, reaching the top where a carrot awaited. Patients performed the Mendelsohn maneuver by attempting to elevate the larynx for 2–3 s to reach the highest point. As the patient exceeded the threshold value of muscle activity, the bunny in the game would climb the hill and reach the top. Then, during the relaxation phase, the patient would release themselves, and the bunny would descend the hill and eat the carrot, completing one exercise cycle. The relaxation period was set to 2 s, and if muscle relaxation was not achieved within 2 s, the game would reset. This approach not only focused on strength but also involved coordination-based tasks. The duration of holding the larynx at the highest point could reach up to a maximum of 5 s, depending on the individual and the treatment process. Like the rose game, the rabbit game provided both visual and auditory feedback.

**Fig. 2:**
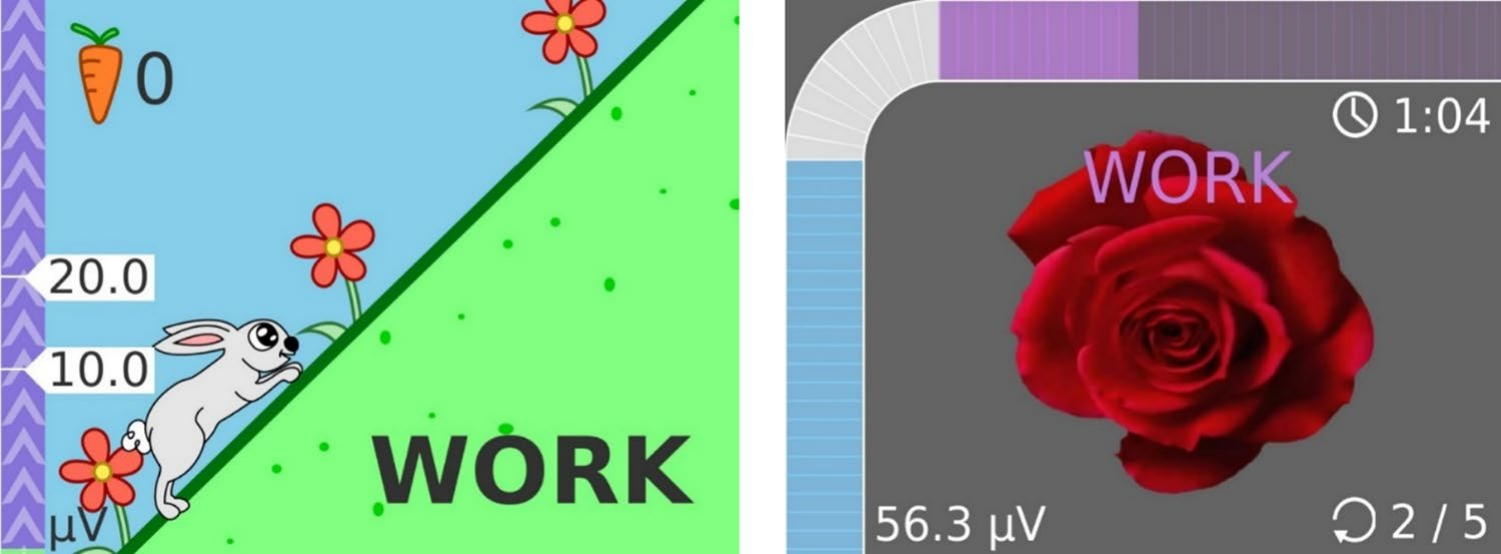
Rose Game and Bunny Game. In the Rose Game, the patient performs an effortful swallow to make the rose wither and releases their muscles to make the rose bloom again. The patient also receives feedback indicating success upon full withering and blooming. In the Bunny Game, the patient performs the Mendelsohn maneuver to help the bunny climb to the top of the mountain. Once the muscles are released, the bunny descends the mountain and eats the carrot. Upon eating the carrot, the patient hears a sound as feedback

To successfully complete each cycle in both games, the patient's muscle activity had to exceed the predetermined threshold value. The exercise duration for each game was 15 min. The threshold value was determined based on the average amplitude of three swallows measured through the Vitalstim Software, and a value above the average was set. Thresholds in the games were initially set as low as possible to build patients' confidence in their swallowing abilities and increase their motivation. The thresholds were gradually increased over time based on the patient's performance, aiming to achieve higher levels of muscle activity (Fig. 2).

### Evaluation Measurements

#### Functional Oral Intake Scale (FOIS)

FOIS was developed by Crary et al. [12] in 2005. It is a two-part scale with a total of 7 levels, indicating the functional oral intake of patients with dysphagia. It is used to determine whether an individual is dependent on tube feeding and to assess the level of oral intake. Items 1 to 3 assess the inability to feed orally, while items 4 to 7 assess the status of oral feeding. It has been evaluated as a suitable tool to determine the change in functional oral intake, particularly in stroke patients. Level 1 represents the worst level, while level 7 indicates the best level. The primary outcome of this RCT is the FOIS.

#### Penetration Aspiration Scale (PAS)

PAS is an eight-item scale developed by Rosenbek et al. [13] in 1996 that provides information about the presence and severity of penetration and aspiration following instrumental videofluoroscopic swallowing assessment. The scale quantitatively determines the presence of penetration and aspiration, making it a reliable and highly clinically applicable tool for all consistencies. Scores are based primarily on the position of the food in the airway and whether it is expelled from the airway. A score of "1" indicates no penetration or aspiration, scores "2–3-4–5" indicate the presence of penetration, while scores "6–7-8" indicate the presence of aspiration.

#### Dysphagia Outcome and Severity Scale (DOSS)

DOSS was developed by O'Neil et al. [14] in 1999. Following videofluoroscopic swallowing evaluation, patients are assessed on a 7-point scale, ranging from normal (7) to severe (1). The scale allows for the classification of dysphagia outcome severity as normal (7), minimal (6), mild (5), mild/moderate (4), moderate (3), moderate/severe (2), and severe (1).

#### Dysphagia Handicap Index (DHI)

DHI is one of the scales used to assess quality of life. Developed by Silbergleit et al. [18], DHI is a 25-item questionnaire that evaluates swallowing difficulty from three aspects: functional, physiological, and emotional. The questionnaire is completed through an interview between the clinician and the patient or their caregiver. Its Turkish validity and reliability were established by Çiyiltepe et al. [19]. Each question has three response options: "never," "sometimes," and "always." "Never" is scored as 0, "sometimes" as 2, and "always" as 4. Scores range from 0 to 100, with higher scores indicating poorer quality of life.

### Statistical Analysis

#### Sample Size Calculation

Using the G*Power 3.1.9.4 software, a power analysis was conducted with the parameters of effect size = 1.1, α = 0.05, and Power (1-β) = 0.80. The minimum required sample size for each group was calculated as 15 participants. Considering a potential drop-out rate of 10%, the number of participants to be included in each group was determined to be 17.

### Data Analysis

Statistical evaluation was performed using IBM SPSS 20.0 (IBM Corp., Armonk, NY, USA). Conformity of the variables to normal distribution was examined by Shapiro–Wilk test. Normally distributed variables were expressed as mean ± SD, and non-normally distributed variables were expressed as median (25th–75th percentile). Categorical variables were presented as frequency (percentage). The difference between the groups was determined by independent samples t-test and Mann–Whitney U test. Wilcoxon signed rank test was used for dependent group comparisons. The relationships between categorical variables were determined by Chi-square analysis. A p value of < 0.05 was considered statistically significant in all analyses.

## Results

**Fig. 3:**
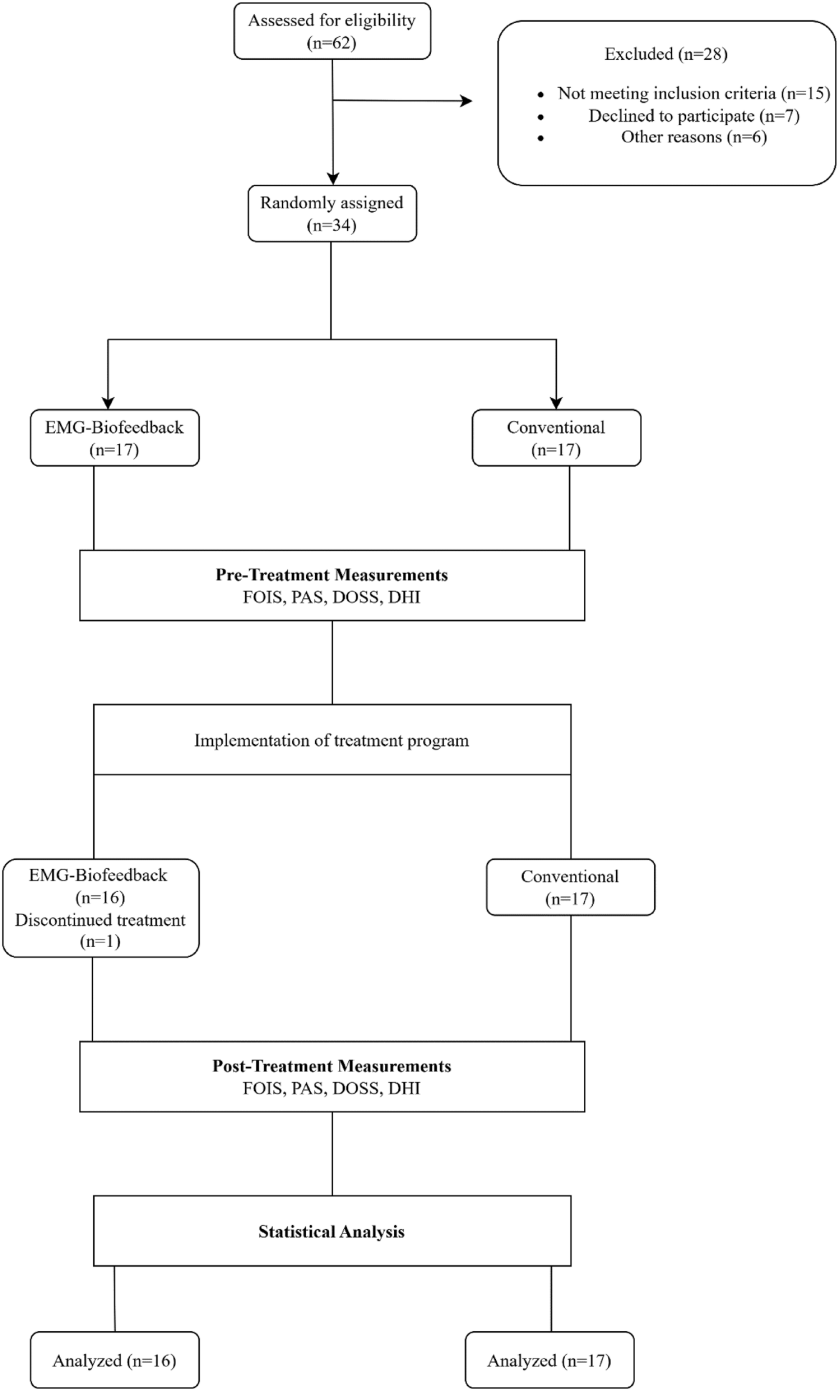
CONSORT flow diagram. *FOIS* functional oral intake scale, *PAS* Penetration aspiration scale, *DOSS* Dysphagia outcome and severity scale, *DHI* Dysphagia handicap index

A total of 34 patients with dysphagia after stroke were included in this study (Fig. 3).

### Clinical and Demographic Features

Key demographic characteristics include patients' age, stroke type, and type of nutrition, which provide an overview of the study population. Baseline scores for cognitive function (MMSE), mobility levels (FAS), and initial swallowing capacity (GUSS) were also recorded. These variables are essential for evaluating the comparability of the study groups and understanding the baseline factors that could influence treatment outcomes.

The participants had a mean age of 61.93 ± 11.88 years, with 66.7% being male. The median stroke duration was 5 months (IQR: 4–6.5), and 90.9% of the participants had ischemic strokes. There was no statistically significant difference between the two groups included in the study in terms of demographic data and clinical features (Table 2).

**Table 2:** Demographic characteristics and clinical data of the patients

	Total patients (n = 33)	Study group (n = 16)	Control group (n = 17)	p
Age, *mean* ± *SD*	61.93 ± 11.88	63.38 ± 11.38	60.59 ± 12.54	0.510*
Sex				1.000***
Female	11 (33.3%)	5 (31.3%)	6 (35.3%)	
Male	22 (66.7%)	11 (68.8%)	11 (64.7%)	
Stroke duration *Median (IQR*)	5 (4/6.5)	5 (3.25/6.75)	5 (4/6.5)	0.817**
Stroke type				1.000***
Ischemic	30 (90.9%)	15 (93.8%)	15 (88.2%)	
Hemorrhagic	3 (9.1%)	1 (6.3%)	2 (11.8%)	
Lesion location				0.398***
Supratentorial	27 (81.8%)	12 (75%)	15 (88.2%)	
Infratentorial	6 (18.2%)	4 (25%)	2 (11.8%)	
Nutrition type				1.000***
Oral	27 (81.8%)	12 (36.3%)	15 (45.4%)	
Tube dependent	6 (18.2%)	4 (12.1%)	2 (6%)	
MMSE *median (IQR)*	24 (24/27)	24.5 (24/28)	24 (24/26)	0.557**
FAS *median (IQR)*	3 (1/ 4)	3 (1.25/4)	2 (0/4)	0.581**
Barthel Index *median (IQR)*	70 (57.5/80)	72.5 (66.25/80)	65 (45/82.5)	0.276**
GUSS *median (IQR)*	11 (8/14)	12 (10/13.75)	10 (8/14.5)	0.657**

*MMSE* Mini mental state examination, *FAS* Functional ambulation scale, *GUSS* Gugging swallowing screen, *SD* Standard deviation, *IQR* Interquartile range

*Independent Sample T-Test

**Mann–Whitney U test

***Chi-square test

### FOIS Outcomes

Before treatment, there was no statistically significant difference in FOIS scores between the two groups. After treatment, statistically significant improvement was observed in FOIS scores in the study group compared to before treatment. In the control group, there was no significant change in post-treatment scores compared to pre-treatment scores. However, there was no statistically significant difference between the groups in terms of post-treatment FOIS scores and the level of change in FOIS scores (Table 3). After treatment, 2 of the 4 patients in the study group who were fed with a feeding tube could be switched to oral feeding. In the control group, both patients who were tube-fed before treatment continued tube feeding after treatment.

**Table 3:** FOIS results of the study and control groups before and after treatment

FOIS	Study group *Median (IQR)*	Control group *Median (IQR)*	p^*^
Before treatment	5.5 (3.5/6)	5 (5/6)	0.986
After treatment	6 (5/6)	6 (5/6)	0.581
Change	0 (0/1)	0 (0/0)	0.309
p**	**0.038**	0.157	

*FOIS* Functional oral intake scale, *IQR* Interquartile range

*p: P value of intergroup analyses (Mann–Whitney U test was used)

**p: P value of within-group analyses (Wilcoxon Signed Rank test was used)

### PAS Outcomes

The PAS score was analyzed separately for semisolid and liquid foods. In inter-group evaluation, there was no statistically significant difference between the two groups in pre-treatment PAS-Liquid scores. In intra-group evaluation, post-treatment values in the study group showed a statistically significant improvement compared to pre-treatment values. However, there was no statistically significant improvement in the control group. There was no statistically significant difference in post-treatment PAS-Liquid scores and the change in PAS-Liquid scores between the groups.

Before treatment, there was no statistically significant difference in PAS-Semisolid scores between the two groups. In intra-group evaluations, post-treatment scores in both groups showed statistically significant improvements compared to pre-treatment scores. In inter-group evaluation, post-treatment PAS-Semisolid scores and the change in PAS-Semisolid scores were higher in the EMG-biofeedback group (Table 4).

**Table 4:** PAS results of the study and control groups before and after treatment

	Study group *Median (IQR)*	Control group *Median (IQR)*	p^*^
PAS-liquid			
Before treatment	5 (4/6.75)	4 (4/6)	0.901
After treatment	4 (2.5/5.75)	4 (4/6)	0.260
Change	0 (− 1/0)	0 (0/0)	0.204
**p	**0.026**	0.180	
PAS-semisolid			
Before treatment	4 (2/5)	3 (2.5/4.5)	0.873
After treatment	2 (1/2)	2 (2/4)	**0.031**
Change	− 1.5 (− 2.75/0.25)	0 (− 1/0)	**0.011**
**p	**0.002**	**0.023**	

*PAS* Penetration Aspiration Scale, *IQR* Interquartile range

*p: P value of intergroup analyses (Mann–Whitney U test was used)

**p: P value of within-group analyses (Wilcoxon Signed Rank test was used)

### DOSS Outcomes

Before treatment, there was no statistically significant difference in DOSS scores between the study and control groups. In intra-group evaluations, post-treatment values showed a statistically significant improvement compared to pre-treatment values in the EMG-biofeedback group. However, there was no significant change in the control group. In inter-group evaluation, the change in DOSS scores was significantly higher in the EMG-biofeedback group (p = 0.004). DOSS results are presented in Table 5.

**Table 5:** DOSS results of the study and control groups before and after treatment

DOSS	Study group *Median (IQR)*	Control group *Median (IQR)*	p^*^
Before treatment	4 (3/4)	4 (2.5/4.5)	0.363
After treatment	4.5 (4/5)	4 (3/5)	0.217
Change	1 (0/2)	0 (0/0)	**0.004**
**p	**0.003**	0.083	

*DOSS* Dysphagia Severity Scale, *IQR* Interquartile range

*p: P value of intergroup analyses (Mann–Whitney U test was used)

**p: P value of within-group analyses (Wilcoxon Signed Rank test was used)

### DHI Outcomes

There was no statistically significant difference between the study and control groups in terms of pre-treatment DHI scores. In intra-group evaluations, post-treatment scores significantly improved compared to pre-treatment scores in both groups. In inter-group evaluation, post-treatment scores were significantly better in the study group. Additionally, the change in DHI-Total scores before and after treatment was greater in the EMG-biofeedback group within the study group (Table 6).

**Table 6:** DHI results of the study and control groups before and after treatment

	Study group *Median (IQR)*	Control group *Median (IQR)*	p^*^
DHI-total			
Before treatment	57 (48/65)	64 (50/75)	0.423
After treatment	41 (32/50)	60 (45/73)	**0.041**
Change	− 12 (− 21.5/− 6)	− 4 (− 6/− 2)	< **0.001**
**p	< **0.001**	**0.001**	
DHI-physical			
Before treatment	24 (18.5/26)	22 (19/30)	0.845
After treatment	17 (12/20)	20 (16/29)	0.087
Change	− 4 (− 8/− 2)	− 2 (− 2/0)	**0.001**
**p	< **0.001**	**0.001**	
DHI-emotional			
Before treatment	19 (14.5/22)	20 (17/24)	0.657
After treatment	13 (8/17.5)	20 (15/22)	**0.037**
Change	− 4 (− 9.5/− 2.5)	− 2 (− 2/0)	< **0.001**
**p	< **0.001**	**0.003**	
DHI-functional			
Before treatment	15 (12/20)	20 (13/24)	0.179
After treatment	13 (8/17.5)	18 (13/23)	**0.041**
Change	− 2 (− 4/− 2)	0 (− 2/0)	**0.005**
**p	**0.001**	0.008	

*DHI* Dysphagia Handicap Index, *IQR* Interquartile range

*p: P value of intergroup analyses (Mann–Whitney U test was used)

**p: P value of within-group analyses (Wilcoxon Signed Rank test was used)

## Discussion

In the present study, both conventional exercises and game-based EMG-biofeedback resulted in improvement in swallowing function and quality of life in post-stroke dysphagia patients. The results also showed that the improvement was greater when exercises were applied through EMG-biofeedback. The most important factor contributing to recovery after a stroke is neuroplasticity [15]. Neuroplasticity is best defined as the brain's and nervous system's ability to change structurally and functionally. Exercise, which promotes neuroplasticity and constitutes a significant part of rehabilitation, increases cerebral blood flow and vascularity, supporting recovery in damaged brain tissue by facilitating dynamic processes in the nervous system. Exercise serves as an effective method in improving motor functions by triggering neuroplasticity through developments in axonal myelination following a stroke, as well as increases in neuronal activity and reinforcement of postsynaptic stimulation [16]. Although the focus and amount of exercise vary greatly from one rehabilitation approach to another, generally, exercise-based swallowing interventions have been shown to improve functional swallowing, minimize or prevent morbidities related to dysphagia, and enhance impaired swallowing physiology [17].

The Mendelsohn maneuver has been used for many years to treat patients with oropharyngeal dysphagia [18]. It has been shown to stimulate the laryngeal reflex, increase the duration of upper esophageal sphincter (UES) opening, prolong laryngeal elevation, and reduce UES peak contraction pressure [19–21]. Effortful swallow, another common therapeutic swallowing exercise, is widely used in dysphagia rehabilitation. It is widely used in dysphagia rehabilitation. Initially designed by Pouderoux and Kahrilas [22], effortful swallow was first recommended for patients with reduced tongue base retraction towards the posterior pharyngeal wall and has been shown to increase tongue root retraction. Subsequently, its various benefits have been demonstrated. The use of effortful swallow exercises alters swallowing biomechanics and affects bolus flow patterns. It is also associated with airway protection [23]. Effortful swallow increases oral floor muscle activity, reduces residue, increases pressure in the pharynx, and decreases UES pressure [24, 25].

Effortful swallow and the Mendelsohn maneuver are examples of task-specific strength training that can meet the criteria for optimizing neuroplasticity when applied intensively with challenging and staged goals, and their combined use is common in dysphagia rehabilitation [26]. The general aim of these maneuvers is to improve bolus passage and residue clearance, ensure airway protection, and increase hyoid movement, laryngeal elevation, and upper esophageal sphincter opening. In a study conducted with ten healthy adults, cortical activity during these exercises was demonstrated by functional MRI. Both effortful swallow and the Mendelsohn maneuvers elicited significantly higher responses in swallowing-related regions compared to normal swallowing [27].

Motor learning is a series of internal processes that lead to changes in performing movements and maintaining them, necessary for acquiring the ability to produce skilled performance [28]. Motor learning is typically acquired through practice or experience [29]. Feedback influencing motor learning can be classified as internal and external. Internal feedback refers to the individual's sensory-perceptual information obtained as a result of the executed movement [28]. External feedback, on the other hand, is information related to the movement in the context of the environment in which it is performed. It is provided through an external source and is usually an additional element to internal feedback sources. External feedback can be non-verbal (auditory, visual, tactile) or verbal (words such as correct, incorrect, etc.) information, or it can be provided in the form of a score or another task success signal by the environment itself. When such information cannot be perceived by the body's sensory systems, external feedback can enhance or replace internal feedback [30]. In patients with neurological disorders, internal feedback systems may be affected by sensory loss. Providing external feedback to these patients can provide increased baseline information regarding movement physiology and thus promote motor learning [31]. Biofeedback is a type of external feedback that provides information about internal physiological events. It provides additional information beyond and above the naturally available information that patients would not normally have access to in real-time. It complements existing internal feedback (e.g., visual, auditory, and proprioceptive feedback) and serves as a "sixth sense" [32]. Biofeedback increases the speed of motor learning, thereby increasing the time efficiency of therapy [33].

EMG-biofeedback is the most widely researched method of biofeedback and appears to be effective in the treatment of many musculoskeletal disorders [34]. In the treatment of post-stroke dysphagia, as in other diseases, the most commonly used feedback method is EMG-biofeedback therapy. Studies in the literature report positive results after the application of task-specific exercises in combination with EMG-biofeedback [35, 36]. In a study, it was reported that all biomechanical events during swallowing showed a strong correspondence with the sEMG signal [37]. The recent emergence of video game systems with motion tracking capabilities has increased interest in video game therapy as a way to increase patient engagement during rehabilitation [38]. Striatal dopamine release during video game play may facilitate cortical plasticity after perceptual learning, and the use of fun games to practice sensory-motor skills may improve patient compliance. Therefore, combining EMG-biofeedback with video games may enable faster and more comprehensive learning during swallowing rehabilitation [39]. For these reasons, we applied game-based EMG-biofeedback therapy instead of graphical EMG-biofeedback. We believe that the addition of games to the treatment contributes to the improvement of physiological parameters related to swallowing.

In this study, the swallowing function of patients was clinically evaluated using the FOIS. FOIS has been widely utilized as an outcome measure in numerous studies on swallowing in the literature. It has been found that the application of EMG-biofeedback in dysphagia patients improves FOIS scores more effectively than traditional exercises [7, 40, 41]. In one study, an improvement in FOIS scores was accompanied by enhanced hyoid bone displacement [42]. In another randomized controlled trial, FOIS scores significantly improved in the EMG-biofeedback group; however, no significant difference was observed in FOIS score changes or post-treatment FOIS scores between the groups [43]. The findings of this study are consistent with the results of our study. FOIS is solely an indicator of feeding status and does not evaluate swallowing function or related physiological parameters. Therefore, in patients with high FOIS scores, treatment effectiveness may be demonstrated through other parameters, but no changes may be observed in FOIS scores. In this study, the median FOIS score was 5.5, and most patients were already orally feeding prior to treatment. This may explain the lack of statistical difference in post-treatment scores and intra-group improvements. To obtain statistically significant results, a larger patient population is needed. Additionally, establishing a specific threshold for FOIS would provide more reliable outcomes.

In this study, PAS scoring was also used as an outcome measure. In the literature, Nordio et al. [7] utilized PAS as an outcome measure and reported that PAS-Semisolid scores improved significantly in the EMG-biofeedback group. These findings are consistent with the results of our study. However, other studies in the literature have shown that while PAS scores improved in the EMG-biofeedback group, this improvement did not reach statistical significance between groups [21, 26, 44]. All these studies were conducted with small patient populations. Although the results are partially contradictory, it can be argued that the relatively larger patient population in our study demonstrates the beneficial effect of EMG-biofeedback on PAS scores more clearly. Additionally, none of these studies utilized a randomized controlled design. We believe that the results obtained in this study are significant in this regard. Another scale used in the present study was DOSS. Looking at the literature, DOSS was the outcome measure in only one study. McCullough et al.[21] showed improvement in DOSS scores with biofeedback, but there was no statistically significant difference in DOSS scores between the biofeedback and control groups. These findings contradict the results of our study. However, the methodologies of both studies are largely different. Two randomized groups received treatment for a two-week period followed by a 2-week period without treatment. According to our findings, EMG-biofeedback therapy improved DOSS scores in post-stroke dysphagia.

DHI was used to assess the quality of life of the patients. A review of the literature reveals that previous studies on this topic have primarily focused on improving swallowing function and related physiological parameters, while neglecting the psychological changes experienced by patients. However, dysphagia is a socially and psychologically devastating condition, and its psychological effects are frequently observed. Eating and swallowing are not merely physical acts of food intake but also encompass social, psychological, and cultural experiences. Quality of life refers to an individual's physical, mental, and social well-being, while health-related quality of life reflects how illness and treatment affect these aspects of life. Therefore, it was important to reveal changes in the quality of life of patients. In the literature, one study reported that DHI improved in both the EMG-biofeedback and control groups; however, interestingly, the improvement was greater in the control group, with no significant differences between the groups [26]. This was a feasibility study with methodological weaknesses, and its findings were noted to require cautious interpretation. In another study, consistent with our findings, quality of life improved more in the biofeedback group [45]. This study is noteworthy due to its larger patient population and stronger methodology. Similarly, according to our results, exercise therapy with EMG-biofeedback improves swallowing-related quality of life more effectively than conventional therapies.

### Strengths and Limitations

The simultaneous evaluation of both swallowing functions and quality of life, along with the prospective randomized controlled design, are the strongest aspects of the present study. Additionally, the study was assessor-blind, with all assessments conducted by a blinded evaluator, and standardized scales and methods were used as primary and secondary outcome measures to assess the effectiveness and safety of swallowing. Furthermore, demographic and clinical characteristics that may affect the results, as well as the pre-treatment values of evaluation parameters, were homogeneously distributed between the two groups. However, this study also has some limitations. The lack of long-term follow-up is the most significant limitation of the study. The lack of long-term follow-up prevented the determination of the sustainable effects of the therapy. Another limitation is the relatively small sample size. The relatively small sample size in the study may have limited the ability to evaluate differences between groups and affected the generalizability of the results. Additionally, the selection of a patient population with specific demographic and clinical characteristics may restrict the applicability of the findings to other patient groups. To overcome these limitations, future research should focus on larger sample sizes, multi-center studies, long-term follow-up protocols, and the inclusion of more diverse patient populations, as well as alternative feedback methods in control groups, to facilitate more comprehensive investigations.

## Conclusion

This is the first prospective randomized controlled trial to investigate the effects of game-based EMG-biofeedback therapy on swallowing function and quality of life in post-stroke dysphagia patients. The results showed that the addition of game-based EMG-biofeedback to conventional therapy had additional positive effects on clinical and radiological parameters. Moreover, improvement in certain parameters was significantly greater in the EMG-biofeedback group compared to that in the control group. Additionally, game-based EMG-biofeedback therapy resulted in a significantly greater improvement in quality of life compared to conventional treatment alone. The results obtained in the present study demonstrate that implementing exercise therapy with game-based EMG-biofeedback enhances the therapeutic effectiveness of exercises. Further randomized controlled trials with larger patient cohorts and different treatment intensities are needed to clearly demonstrate the efficacy of game-based EMG-biofeedback treatment.

## Data Availability

Data are available from the corresponding author upon reasonable request.
