# Correction: Efficacy of Game-Based EMG-Biofeedback Therapy in Post-Stroke Dysphagia: A Randomized Controlled Trial

**DOI:** 10.1007/s00455-025-10901-8

**Published:** 2025-10-27

**Authors:** Bülent Alyanak, Murat İnanır, Selime Ilgın Sade, Serkan Kablanoğlu

**Affiliations:** 1 Department of Physical Medicine and Rehabilitation, Kocaeli University Faculty of Medicine, Kabaoğlu, Baki Komsuoğlu Bulvarı No:515, Umuttepe, İzmit, Kocaeli 41001 Turkey; 2 Institute of Health Sciences, Kocaeli University, Kabaoğlu, Baki Komsuoğlu Bulvarı No:515, Umuttepe, İzmit, Kocaeli 41001 Turkey

**Correciton to: Dysphagia**

10.1007/s00455-025-10819-1

In this article, it was determined that an incorrect version of the manuscript had been published. This occurred due to a production error that resulted in the publication of a version containing incomplete revisions.

The necessary corrections have been made, and the published version has been updated to accurately reflect the correct content. These corrections involve formatting and textual changes; however, they do not affect the results, data interpretation, or conclusions of the study.

The original article has been corrected.

